# Non-CpG sites preference in G:C > A:T transition of *TP53* in gastric cancer of Eastern Europe (Poland, Romania and Hungary) compared to East Asian countries (China and Japan)

**DOI:** 10.1186/s41021-022-00257-y

**Published:** 2023-01-04

**Authors:** Hiroko Natsume, Kinga Szczepaniak, Hidetaka Yamada, Yuji Iwashita, Marta Gędek, Jelena Šuto, Keiko Ishino, Rika Kasajima, Tomonari Matsuda, Felix Manirakiza, Augustin Nzitakera, Yijia Wu, Nong Xiao, Qiong He, Wenwen Guo, Zhenming Cai, Tsutomu Ohta, Tıberiu Szekely, Zoltan Kadar, Akiko Sekiyama, Takashi Oshima, Takaki Yoshikawa, Akira Tsuburaya, Nobuhito Kurono, Yaping Wang, Yohei Miyagi, Simona Gurzu, Haruhiko Sugimura

**Affiliations:** 1 Department of Tumor Pathology, Hamamatsu University School of Medicine, 1-20-1 Handayama, Higasi-ku, Hamamatsu, Shizuoka 431-3192 Japan; 2 Medical University of Warsaw, 1B Banacha Street, Warsaw, Poland; 3 Medical University of Lublin, ul. Radziwiłłowska 11, wew, 5647 Lublin, Poland; 4 Department of Oncology, Clinical Hospital Centre Split, Split, Croatia; 5 The Center for Cancer Genome Medicine, Kanagawa Cancer Center, 2-3-2 Nakao, Asahi-ku, Yokohama, Kanagawa 241-8515 Japan; 6 Molecular Pathology and Genetics Division, Kanagawa Cancer Center Research Institute, 2-3-2 Nakao, Asahi-ku, Yokohama, 241-8515 Japan; 7 Research Center for Environmental Quality Management, Kyoto University, 1-2 Yumihama, Otsu, Shiga 520-0811 Japan; 8 Lujiang People Hospital, 32 Wenmingzhong Road, Lujiang, Hefei, 231501 China; 9 Jiangsu Key Laboratory of Molecular Medicine, Nanjing University School of Medicine, Nanjing, 210093 China; 10 Department of Pathology, The Second Affiliated Hospital of Nanjing Medical University, Nanjing, 210003 China; 11 Department of Immunology, Key Laboratory of Immune Microenvironment and Diseases, Nanjing Medical University, Nanjing, 211166 China; 12 Department of Physical Therapy, Faculty of Health and Medical Sciences, Tokoha University, 1230 Miyakoda-cho, Kita-ku, Hamamatsu, Shizuoka 431-2102 Japan; 13 Department of Pathology, George Emil Palade University of Medicine, Pharmacy, Sciences and Technology, Targu Mures, Ghe Marinescu 38 Street, 540139 Targu Mures, Romania; 14 Department of Oncology, George Emil Palade University of Medicine, Pharmacy, Sciences and Technology, Targu Mures, Ghe Marinescu 38 Street, 540139 Targu Mures, Romania; 15 Department of Clinical Laboratory, Kanagawa Cancer Center, 2-3-2 Nakao, Asahi-ku, Yokohama, Kanagawa 241-8515 Japan; 16 Department of Gastrointestinal Surgery, Kanagawa Cancer Center, 2-3-2 Nakao, Asahi-ku, Yokohama, Kanagawa 241-8515 Japan; 17 Department of Gastric Surgery, National Cancer Center Hospital, 5-1-1 Tsukiji, Chuo-ku, Tokyo, 104-0045 Japan; 18 Department of Surgery, Ozawa Hospital, 1-1-17, Honcho, Odawara, Kanagawa 250-0012 Japan; 19 Department of Chemistry, Hamamatsu University School of Medicine, 1-20-1 Handayama, Higashi-ku, Hamamatsu, Shizuoka 431-3192 Japan; 20 Sasaki Foundation Sasaki Institute, 2-2, KandaSurugadai, Chiyoda-ku, Tokyo, 101-0062 Japan

**Keywords:** Gastric cancer, TP53, Molecular epidemiology, Mutation spectrum, Mutation signature, Gene-environmental interaction, Adductomics, Geographic, Pathology

## Abstract

**Aim:**

Mutation spectrum of *TP53* in gastric cancer (GC) has been investigated world-widely, but a comparison of mutation spectrum among GCs from various regions in the world are still sparsely documented. In order to identify the difference of *TP53* mutation spectrum in GCs in Eastern Europe and in East Asia, we sequenced *TP53* in GCs from Eastern Europe, Lujiang (China), and Yokohama, Kanagawa (Japan) and identified the feature of *TP53* mutations of GC in these regions.

**Subjects and method:**

In total, 689 tissue samples of GC were analyzed: 288 samples from East European populations (25 from Hungary, 71 from Poland and 192 from Romania), 268 from Yokohama, Kanagawa, Japan and 133 from Lujiang, Anhui province, China. DNA was extracted from FFPE tissue of Chinese, East European cases; and from frozen tissue of Japanese GCs. PCR products were direct-sequenced by Sanger method, and in ambiguous cases, PCR product was cloned and up to 8 clones were sequenced. We used No. NC_000017.11(hg38) as the reference sequence of *TP53*. Mutation patterns were categorized into nine groups: six base substitutions, insertion, deletion and deletion-insertion. Within G:C > A:T mutations the mutations in CpG and non-CpG sites were divided. The Cancer Genome Atlas data (TCGA, ver.R20, July, 2019) having somatic mutation list of GCs from Whites, Asians, and other ethnicities were used as a reference for our data.

**Results:**

The most frequent base substitutions were G:C > A:T transition in all the areas investigated. The G:C > A:T transition in non-CpG sites were prominent in East European GCs, compared with Asian ones. Mutation pattern from TCGA data revealed the same trend between GCs from White (TCGA category) vs Asian countries. Chinese and Japanese GCs showed higher ratio of G:C > A:T transition in CpG sites and A:T > G:C mutation was more prevalent in Asian countries.

**Conclusion:**

The divergence in mutation spectrum of GC in different areas in the world may reflect various pathogeneses and etiologies of GC, region to region. Diversified mutation spectrum in GC in Eastern Europe may suggest GC in Europe has different carcinogenic pathway of those from Asia.

**Supplementary Information:**

The online version contains supplementary material available at 10.1186/s41021-022-00257-y.

## Introduction

Gastric cancer (GC) is the fifth most common cancer and the third most common cause of cancer-related mortality (https://gco.iarc.fr/today/home) [1]. GC incidence differs with the region, and its prevalence changes over time [1]. In most western countries, the occurrence of GC is declining but remains a substantial cause of cancer-related deaths. East Asian countries, such as China and Japan, have the highest incidence of GC in the world. Meanwhile, several countries or areas in Europe, such as Italy, Spain, and East Europe [2], are known to have a considerably higher incidence of GC than other parts of Europe [3].

The time trends and geographic variation reflect the difference in causative factors of GC, such as differences in environmental factors, lifestyle, infection, traditional foods, and salty diet, as well as the genetic structure of individual populations.

The spectrum of somatic *TP53* mutations, the most prevalent gene mutations found in human tumors, has provided important clues for environmental carcinogenesis [4]. In the last 40 years, the mutation spectrum linking some aspects of human environmental carcinogenesis has been explored by studying *TP53* mutations in various human cancers in specific settings, such as tobacco smoking, UV damage, and aflatoxin exposure [4]. For example, cancer mutations related to etiological pathways include the G to T transition in tobacco smokers’ cancers, CC > TT in skin cancer via ultraviolet (UV) irradiation, and *TP53* (AGG > AGT, p.Arg249Ser) in aflatoxin B-related hepatocellular carcinoma. These specific mutation spectra are currently being recapitulated as mutation signatures based on next-generation sequencing data, including single base substitution (SBS) 4, SBS 7, and SBS24 corresponding to tobacco, UV, and aflatoxin B, respectively [5]. However, the populations used for generating these data are known to be biased, and the information on tumors from some countries is less scrutinized than that in several urbanized areas [6–8].

The *TP53* database was created from voluminous mutation spectrum data in various cancers collected from different populations around the world. The IARC *TP53* database by the NCI and other published reports indicated that the *TP53* mutation observed in GC mainly involves G:C > A:T transitions [9].

Although there are anecdotal reports on GC mutations from Poland [10, 11], information on GC in East Europe is generally sparse. There is often missing or vague ancestry or cultural information regarding subjects of European or white descent in several databases. In The Cancer Genome Atlas (TCGA) database, *TP53* mutations in GC have mainly been found in Asians, Whites, Blacks, American Indians, Alaskan Natives, and Hawaiian natives.

In this report, for the first time, we characterized considerable numbers of *TP53* mutations in GC samples from East European countries (Romania, Poland, and Hungary) and compared these with mutation spectra observed in GC samples from East Asian countries (China and Japan).

## Materials and methods

### Samples

In total, 689 GC tissue samples from three populations worldwide were analyzed: 288 samples from the European population (25 from Hungary, 71 from Poland, and 192 from Romania), 268 from Japan, and 133 from China. The clinical profiles of the patients are summarized in Table 1. Histological classification was conducted according to the Lauren classification by attending pathologists in each area; thus, the sizes and numbers of blocks (coverage of pathological examination) varied among the three regions.

**Table 1:** Clinicopathological profiles of patients with GC from Eastern Europe, China, and Japan

	Eastern Europe		China		Japan		
	*n* = 188		*n* = 133		*n* = 268		*p*-value^c^
**Age**							
Mean	62.6		61.2		63.7		0.02
Range	22-98		40-77^b^		25-85		
	n	%	n	%	n	%	*p*-value^d^
**Sex**							
Male	190	66.2	96	74.4	166	69.7	
Female	97	33.8	33	25.6	72	30.3	
Unknown	1^a^		4		30		
**Subtype**							
Diffuse	127	44.1	69	51.9	150	56.0	2 × 10^−5^
Intestinal	161	55.9	60	45.1	83	31.0	
Others or unknown			4	3.0	35	13.1	

^a^The sex information was not available in one case from Eastern Europe

^b^The ages of two Chinese cases (one male, one female) were unknown

^c^ *P*-value of the t-test for the ages between China and Japan

^d^ *P-*value of the χ^2^ test for histological subtypes among the three groups: Eastern Europe, China, and Japan

### DNA extraction

The pathology archives of GC formalin-fixed paraffin-embedded tissues (FFPE) were collected from three countries in Eastern Europe and Lujiang County, Anhui Province, China. Fresh GC tissues were obtained from the Pathology Department of Kanagawa Cancer Center, Yokohama. DNA was extracted from the FFPEs using the QIAamp DNA FFPE Tissue Kit (Qiagen, Valencia, CA, United States) [12, 13], while DNA extraction from fresh frozen tissue was performed according to a previously published report [14].

### PCR amplification and sequencing

*TP53* gene sequencing was performed by direct sequencing using a polymerase chain reaction (PCR) product amplified using respective primer sets for each exon. Fragments covering exons 2 to 11 and the boundary regions of the *TP53* gene were amplified via PCR using the HotStarTaq DNA polymerase (Qiagen). The PCR products were purified with Exo-SAP-IT (Thermo Fisher Scientific, Waltham, MA, USA) and sequenced via the Sanger method using the BigDye Terminator Cycle Sequencing Reaction Kit, ver.3.1 and ABI 3130xL Genetic Analyzer (Thermo Fisher Scientific). PCR products exhibiting multiple bands were sequenced after subcloning them into a pGEM-T Easy vector system (Promega, Madison, WI, USA). Up to eight clones were sequenced, particularly upon confirming the presence of insertion/deletion mutations. The primers used are listed in Table S1. Primer design was performed based on the *TP53* reference sequence (Accession No. NC_000017.11(hg38 and GRCh38)). For the DNA samples from FFPEs that were difficult to amplify, and when amplification was not successful, the primer designs were modified to amplify different segments of *TP53*. The resulting sequences were assigned to the reference sequences. DNA mutations were described according to the international guidelines for gene nomenclature [15]. In this experiment, the DNA sequences covered were exons 4 to 9 in samples from China, exons 4 to 8 in samples from Japan, and exons 2 to 11 in those from Eastern Europe.

### Categorization of *TP53* mutations

*TP53* mutation patterns were categorized as follows: G:C > A:T, A:T > G:C, G:C > C:G, G:C > T:A, A:T > C:G, A:T > T:A, deletion, deletion-insertion, insertion, and splice-site mutations in exons 4 to 8. G:C > A:T mutations were subclassified based on their localization in CpG or non-CpG sites. We also grouped *TP53* mutations found here according to the following hotspots proposed by Hainaut P [16]: c.524G > A (R175H), c.586C > T (R196*), c.637C > T (R213*), c.659A > G (Y220C), c.733G > A (G245S), c.742C > T (R248W), c.743G > A (R248Q), c.818G > A (R273H), c.817C > T (R273C), c.844C > T (R282W), c.916C > T (R306*), and c.1024C > T (R342*), in which 11 of these 12 hotspots were localized in exons 4 to 8.

### Distribution of *TP53* mutations

A distribution map of *TP53* mutations was drawn using cBioPortal MutationMapper (https://www.cbioportal.org/mutation_mapper) [17] to determine the difference between the distribution of G:C > A:T at CpG and non-CpG sites.

### Percentage of base substitutions in Caucasian and Asian cases in the TCGA dataset

Information on somatic mutations except for synonymous mutations in the *TP53* gene was downloaded for Caucasian (*n* = 278) and Asian (*n* = 89) populations from the Stomach Adenocarcinoma (TCGA, PanCancer Atlas) data in cBioPortal. The downloaded dataset included 148 Caucasian and 47 Asian patients with intrasomatic mutations. The percentage of base substitution patterns obtained for each race was determined. Since the nucleotide sequence files were not available, we extracted the patterns of G > A or C > T gene mutations to determine if they were in the CpG region, and the sequences of the mutations were compared against the reference genome (GRCh38) by extracting the sequences before and after the base substitution. Base substitutions at the splice site or region were preferentially classified as splice mutations.

### Mutational signature analysis

Substitutions in the coding sequence were determined from the somatic *TP53* mutations. Ninety-six substitution types and sequence contexts were counted for each population. The percentage of each of the 96 substitution types was calculated from the total number of substitutions in each population. The SBS for each population was estimated using Signal (https://signal.mutationalsignatures.com) [18].

### Ethics

This study was a retrospective, anonymous, and non-intervention study, and informed consent from the patients was waived. The research plan was agreed upon by all researchers and approved by the IRB of the Hamamatsu University School of Medicine (G-260 and 20-110), Kanagawa Cancer Center, and the Ethical Committee of the University of Medicine and Pharmacy of Targu-Mures, Romania (Agreement no. 124/28.07.2016).

### Statistical analysis

Statistical analyses were performed using the chi-square test, t-test, and Fisher’s exact test with JMP, ver.11.

## Results

### TP53 mutations and clinicopathological attributes

Pathological findings showed that the intestinal type of GC was predominant in Eastern Europe. Diffuse-type GCs were more prevalent in Japanese subjects than in those from Eastern Europe. Chinese samples showed almost equal proportions of intestinal and diffuse types. There were significant differences in the histological types of GC among the three areas (χ^2^ test, *p* = 2 × 10^− 5^). Japanese patients were older than Chinese patients (t-test, *p* = 0.02). There were no significant differences in sex among the three populations (Table 1).

A total of 689 genomic samples were successfully analyzed for *TP53*-sequencing. Among them, 285 samples (41%) had *TP53* mutations, and 404 (59%) were wild-type. The ratios of mutated cases were 29.5% (85/288), 57.1% (76/133), and 46.3% (124/268) in Eastern Europe, China, and Japan, respectively (χ^2^-test, *p* = 7 × 10^− 8^, Table 2). Based on the histological type, the mutation ratios of both intestinal and diffuse-type GCs were approximately 30% in Eastern Europe. In East Asian groups, the mutation ratios were 40-60% in both histological types, which were relatively higher than those in Eastern Europe. The *TP53* mutation prevalence was more than 50% in both histological types in Chinese subjects, while prevalence was more than 50% only in the intestinal type in Japanese subjects (Table S2). The prevalence of *TP53* mutations differs among areas. The prevalence of *TP53* mutations in the available exon sequences was significantly different (χ^2^ test, *p* = 7 × 10^− 8^) among the three regions; when the prevalence in Japan and China were combined (as East Asia), the prevalence was greater in East Asia than in Eastern Europe (χ^2^ test, *p* = 9 × 10^− 8^).

**Table 2:** *TP53* mutation status in Eastern Europe, China, and Japan (exons 4 to 8)

*TP53* mutation	Eastern Europe		China		Japan		*P*-value^a^	*P*-value^b^
	n	%	n	%	n	%		
Mutant (*n* = 285, 41.4%)	85	29.5	76	57.1	124	46.3	7 × 10^−8^	9 × 10^−8^
Wild type (*n* = 404, 58.6%)	203	70.5	57	42.9	144	53.7		

^a^ *P*-value of the χ^2^ test for the *TP53* mutation prevalence among the three groups: Eastern Europe, China, and Japan

^b^ *P-*value of the χ^2^ test for the *TP53* mutation prevalence between Eastern Europe and East Asia

### Distribution of the mutations

**Fig. 1:**
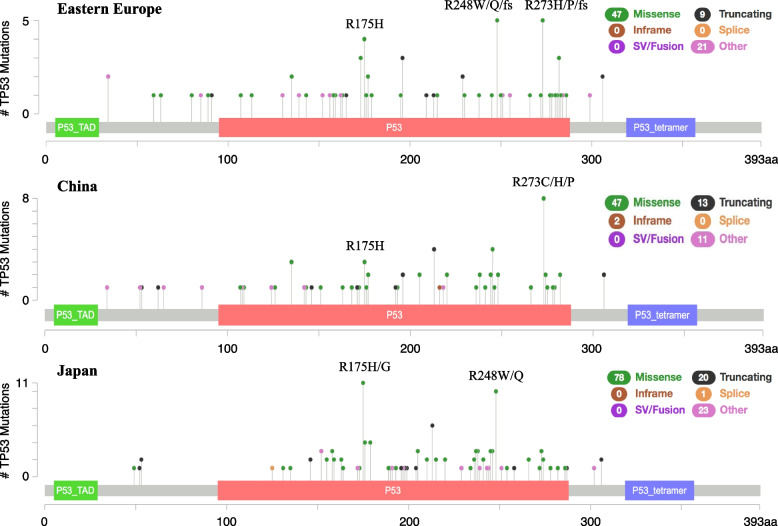
Localization of *TP53* mutations in GC samples from Eastern Europe, China, and Japan (exons 4 to 8). Mutation-distribution maps were created using the cBioPortal mutation mapper (http://www.cbioportal.org/mutation_mapper). Black dots indicate truncating mutations (nonsense and frameshift mutations). Missense mutations were prevalent in all three groups. Some mutation assemblies, including R175H/G and R273C/H/P, were observed in 5-10 cases in each group. P53_TAD, TP53 transcriptional activation domain; P53, TP53 DNA-binding domain; P53 tetramer, TP53 tetramer domain

Lollipop plots (cBioPortal) for the three areas are presented. Mutation-accumulated codons, such as R175H/G, R248W/Q, and R273C/H/P, were observed in each population in five to ten cases (Fig. 1). G:C > A:T mutations at the non-CpG sites of *TP53* were relatively evenly distributed among the three groups. Those at CpG sites involved several mutation assemblies, such as R175H and R248W/Q in Eastern Europe and Japan, and R273C/H in China (Supplementary Fig. S1). These were consistent with hotspot mutations at the CpG sites.

**Fig. 2:**
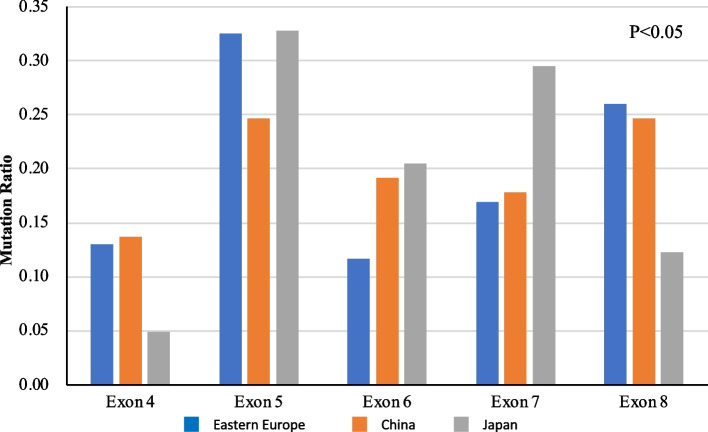
*TP53* mutation assemblies in each exon (exons 4 to 8) in GC samples from Eastern Europe, China, and Japan. *TP53* mutation assemblies in each exon (exons 4 to 8) are shown as ratios of the number of GC mutations in each exon to the total number of mutations in exons 4 to 8 in each group. The blue, orange, and gray bars indicate the *TP53* assembly of each exon in GC samples from Eastern Europe, China, and Japan, respectively. In Eastern Europe, *TP53* mutations were particularly noted in exons 5 and 8. The prevalence of exon 4 mutations was low in Japanese patients. In Chinese patients, the mutations were relatively evenly distributed among exons 4 to 8. The exon distribution of *TP53* mutations was significantly different in Eastern Europe, China, and Japan (χ^2^ test for three regions by five exons; *p* < 0.05)

Mutations in exons 4 to 8 in the three populations are shown in Fig. 2 and Table S3. In this study, 272 (94.4%) out of 285 mutations were found in exons 4 to 8. In Japanese patients, only this coding region was sequenced. Meanwhile, 13 mutations outside these exons were found in samples from Eastern Europe and China and sequenced in exons 2, 3, 9, 10, and 11. As shown in Fig. 2, mutations were the most prevalent in exons 4 to 8 in all regions.

### Mutation type

**Fig. 3:**
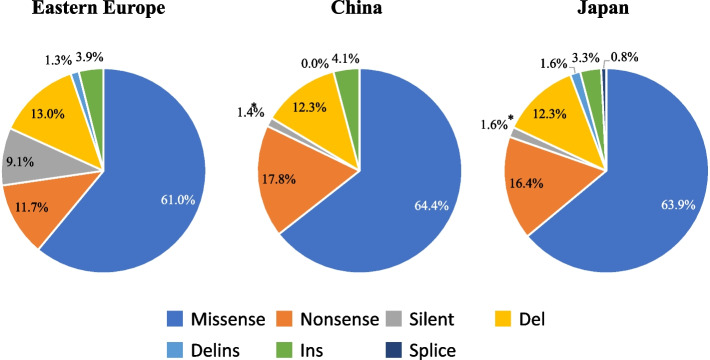
*TP53* mutation types in GC samples from Eastern Europe, China, and Japan (exons 4 to 8). Missense mutations were the most prevalent type (60% of all the mutations) in all the areas. The pie graphs show the percentages of the mutation functions, including missense (blue), nonsense (orange), and silent mutations (gray), deletions (del) (yellow), deletion-insertion (delins) (light blue), insertion (ins) (light green), and splice site mutations (dark blue) in *TP53* in GC samples from Eastern Europe, China, and Japan. All three groups showed similar ratios for each type of mutation. The prevalence of silent mutations (grey) was significantly different between Europe and Asia (*p* < 0.01). * Statistically significant difference (*p* < 0.05)

Missense mutations were predominant in all three regions (Fig. 3, Table S4); the second most common mutation was a nonsense mutation. The ratios of missense and nonsense mutations were approximately 60-65% and 10-18%, respectively, in all three groups (Fig. 3, Table S4). As for histological types, missense mutations were approximately 60-70% in both intestinal and diffuse-type GCs in all three groups. The total ratios of nonsense, silent, and deletion mutants were 25-40% in both intestinal and diffuse types in all three groups. Silent mutations were absent in the diffuse-type GC in China (χ^2^ test, *p* < 0.01) (Supplementary Fig. S2, Table S4).

### Mutation spectrum of *TP53*

**Fig. 4:**
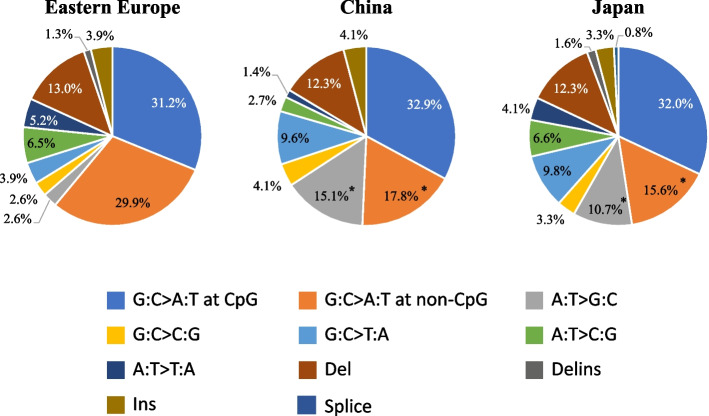
*TP53* mutation spectra in GC samples from Eastern Europe, China, and Japan. The *TP53* mutations in the spectrum were classified into six types of single nucleotide substitutions, as well as deletions (del), insertions (ins), deletion-insertion (delins), and splice mutations. The G:C > A:T transitions were subdivided into G:C > A:T at CpG and non-CpG sites. Each spectrum is shown in the pie graph as follows: G:C > A:T at CpG sites (blue), G:C > A:T at non-CpG sites (orange), A:T > G:C (gray), G:C > C:G (yellow), G:C > T:A (light blue), A:T > C:G (light green), A:T > T:A (dark blue), del (brown), delins (dark gray), ins (light brown), and splice mutations (light navy). The percentages of G:C > A:T transitions at CpG sites were almost equivalent in Eastern Europe, China, and Japan. Those at non-CpG sites showed a significantly higher percentage in Eastern Europe than in East Asian countries. The prevalence of G:C > A:T at non-CpG sites (orange) and A:T > G:C (gray) in Asia was also significantly different from that in Eastern Europe (*p* < 0.05). * Statistically significant difference (*p* < 0.05)

Among the six types of nucleotide alterations, G:C > A:T transition showed the highest frequency of 61.1, 50.7, and 47.6% in exons 4 to 8 in Eastern Europe, China, and Japan, respectively (Fig. 4).

When G:C > A:T mutations were divided into CpG and non-CpG sites, we discovered that G:C > A:T mutations at non-CpG sites were relatively more prevalent in Eastern Europe than in China and Japan. The prevalence of G:C > A:T in non-CpG sites among all the mutations was significantly different in Eastern Europe (29.9%), China (17.8%), and Japan (15.6%) (χ^2^ test, *p* = 0.04) (Fig. 4, Table S5). Although it was impossible to determine whether the prevalence of the different mutations reflects an increase in one type of mutation, a decrease in another, or a combination of the two, the A:T > G:C mutation was mainly found in GC samples from East Asia (χ^2^ test, *p* = 0.03) (Fig. 4). The prevalence of G:C > T:A was 3.9, 9.6, and 9.8% in Eastern Europe, China, and Japan, respectively. The prevalence in Asian countries was higher than that in Eastern Europe; however, there was no significant difference (Table S5).

G:C > A:T mutations were predominant in both intestinal and diffuse types in all three groups, representing 45-70% of all mutations in each group. The prevalence was prominently high (69.6%) in the diffuse type of GC in Eastern Europe, in which the prevalence of G:C > A:T at non-CpG sites was significantly higher (30.3%) (*p* = 0.04) than in East Asian countries. On the other hand, A:T > G:C showed a significantly higher prevalence in China and Japan than in Eastern Europe, especially in diffuse-type GCs (Supplementary Fig. S3, Table S5).

### Results of the TCGA data set

**Fig. 5:**
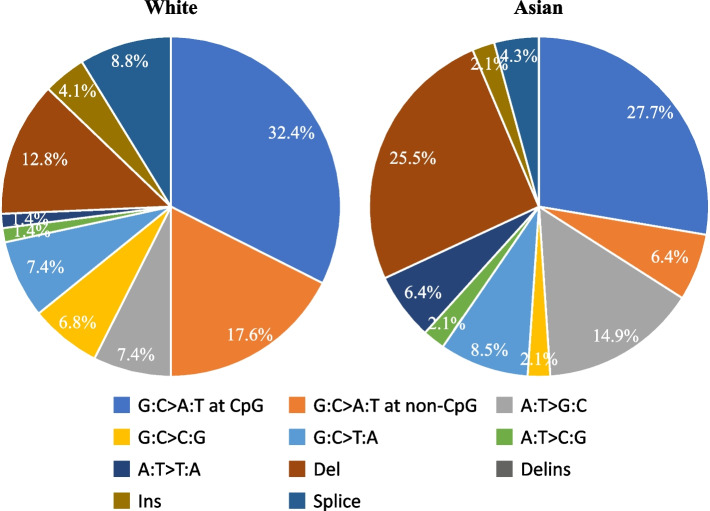
*TP53* mutation spectra in Caucasian and Asian populations in TCGA. The *TP53* mutations in the spectrum were classified into six types of single nucleotide substitutions, as well as deletions (del), insertions (ins), deletion-insertion (delins), and splice mutations. The G:C > A:T transition was subdivided into G:C > A:T at CpG and non-CpG sites. Each spectrum is shown in the pie graph as follows: G:C > A:T at CpG sites (blue), G:C > A:T at non-CpG sites (orange), A:T > G:C (gray), G:C > C:G (yellow), G:C > T:A (light blue), A:T > C:G (light green), A:T > T:A (dark blue), del (brown), delins (dark gray; 0% in both populations), ins (light brown), and splice mutations (light navy)

TCGA categorizes data from different races, including White, Black, Asian, American Indian/Native Alaskan, and Native Hawaiian/ Other Pacific Islanders in the United States. It does not discriminate between Chinese and Japanese; both are designated as “Asian”. The findings are consistent even though the categorization of “Whites” and “Asians” may not be completely the same as our categorization of “East Europeans” and “East Asians”. The G:C > A:T mutations at non-CpG sites were more prevalent in the “White” population than in the Asian population (17.6% vs. 6.4%) (Fig. 5 and Supplementary Fig. S4).

### Mutational signature analysis of *TP53*

Mutational signatures were generated from 63, 61, and 101 substitutions from samples from Eastern Europe, China, and Japan, respectively. The estimated SBS values were SBS1 and SBS5 in Eastern Europe and SBS1 in both China and Japan (Supplementary Fig. S5). The percentage of T > C substitutions appeared to be higher in China and Japan than that in Eastern Europe, although the difference was not significant due to the small number of mutations. A similar trend was observed in both Whites and Asians in TCGA.

### Mutations in the hotspots proposed by Hainaut et al.

The mutations detected here were assigned to recently proposed hotspots [16]. According to Hainaut et al.’s proposal, the mutations in 10 out of 11 hotspots in exons 4 to 8 are G:C > A:T mutations at CpG sites. In this study, the G:C > A:T mutations at the CpG sites in samples from all three areas corresponded to these hotspots. However, the hotspot distribution of these mutations, whose prevalence is more than 5%, appeared different among the three areas. For example, R213* mutations, the most common in the US database, were prevalent in East Asia but not in Eastern Europe, while R175H was shared in all regions. The prevalence of R248Q, also common in U.S. patients, was 5.7% in Japan; that of R273C was 5.5% in China. The prevalence of R248W was 3.9, 0, and 2.5% in Eastern Europe, China, and Japan, respectively. The prevalence of R273H, common in U.S. patients, was 3.9, 4.1, and 1.6% in Eastern Europe, China, and Japan, respectively. To summarize, ≥80% of G:C > A:T mutations belong to the “hot spot mutations” in all three groups. In particular, all mutations in the Chinese population were assembled into hotspots (Table S6).

### Newly identified mutations

Dozens of previously reported mutations were identified in this study, and their functional significance presumed by the software is shown in Table 3.

**Table 3:** Newly identified *TP53* mutations

Population	cDNA description	Protein description	Mutation function	Location
Hungary	c.97-39G > A	intron	substitution	Intron 3
	c.99_100insGTCC	p.(Pro34Valfs*10)	ins	Exon 4
Poland	c.484_488delinsC	p.(Ile162Profs*7)	delins	Exon 5
	c.672 + 83 T > C	intron	substitution	Intron 6
Romania	c.412_422del	p.(Ala138Profs*7)	del	Exon 5
	c.450_478del	p.(Pro151Glyfs*20)	del	Exon 5
	c.622_623insGATA	p.(Asp208Glyfs*2)	ins	Exon 6
	c.672 + 3C > T	p.?	substitution	Intron 6
	c.782 + 23G > A	p.?	substitution	Intron 7
	c.*1C > T	p.(=)	substitution	3′-UTR (Exon 11)
China	c.323_339del	p.(Gly108Valfs*35)	del	Exon 4
	c.98_99insAGTC	p.(Pro34Valfs*10)	ins	Exon 4
	c.255_265del	p.(Ala86Leufs*59)	del	Exon 4
	c.369_375del	p.(Cys124Thrfs*44)	del	Exon 4
	c.195_235del	p.(Arg65Serfs*70)	del	Exon 4
	c.646_647insTAG	p.(Val218dup)	ins	Exon 6
	c.729_732del	p.(Gly244Alafs*2)	del	Exon 7
	c.820_825dup	p.(Val274_Cys275dup)	dup	Exon 8
Japan	c.99_112del	p.(Pro34Serfs*4)	del	Exon 4
	c.503_541del	p.(His168_Glu180del)	del	Exon 5
	c.514_532del	p.(Val172Thrfs*69)	del	Exon 5
	c.449_459del	p.(Thr150Argfs*27)	del	Exon 5
	c.473_474delinsCT	p.(Arg158Pro)	delins	Exon 5
	c.576_599del	p.(Gln192_Asn200delinsHis)	del	Exon 6
	c.588_589del	p.(Val197Glyfs*11)	del	Exon 6
	c.755_757delinsCT	p.(Leu252Profs*93)	delins	Exon 7
	c.776_782 + 1dup	p.?	splice site^a^	Intron 7
	c.97-23_97-13del	p.?	splice site^b^	Intron 3

^a^The duplicated region is involved in splicing the consensus sequence in the 5′-region of the intron

^b^The deleted region is involved in the predicted splicing consensus sequence for the branching site and the subsequent polypyrimidine tract in the intron

## Discussion

Our study revealed different *TP53* mutation profiles in GCs among populations from three regions in the world. All three populations studied here showed the G:C > A:T transition as the most common mutation profile, which is consistent with previous data [9]. Notably, the G:C > A:T mutation at non-CpG sites was more prevalent in GC cases from Eastern Europe than in those from China and Japan. The prevalence of the A:T > G:C mutation was significantly higher in GC cases from East Asia than in those from Eastern Europe. Although studies on the genetic changes in GC cases in Europe are scarce, Palli et al. sequenced the *TP53* gene in GC samples from Florence, Italy, which they claimed to be a “high prevalence area” [19]. Among the 105 cases, 33 mutations were detected, of which 19 had G:C > A:T transitions at CpG sites, and the remaining 14 had transversions, deletions, and transitions at non-CpG sites. Hongyo et al. summarized the published *TP53* mutations in GCs in their *TP53* mutation data in Florence, Italy [20].

Recently, *TP53* mutations in GC in Poland have been reported [10, 11]; among these, four of eight G:C > A:T transitions were documented at non-CpG sites. Our findings were consistent with our analysis of the TCGA data set even though the categorization of “Whites” and “Asians” in TCGA may not be completely the same for Europeans and East Asians in this study. A comprehensive analysis of genetic ancestry shows that the frequency of somatic *TP53* mutations differs among ethnicity [21, 22]. The genetic ancestry and geographical differences described here are not the same, and these studies were not analyzed for GC. Our findings also verify a previous observation in Italy [20] and the presumption of several authors [19, 23] that the pathogenesis of GC in Eastern Europe and East Asia could be different.

The higher frequency of G:C > A:T mutations at CpG sites in GC in Asia probably reflects chronic inflammation, specifically, chronic gastritis caused by chronic *H. pylori* infection [24–27]. Chronic inflammation induces spontaneous deamination of 5-methyl cytosine at CpG sites [28, 29]. G:C > A:T mutations at CpG sites reflect inflammation-mediated carcinogenesis [30–32]. Inflammation-induced DNA methylation via DNMT1 and DNMT3 activation was also investigated using the cytotoxin-associated gene A of *H. pylori* (CagA) [33]. Recently, Ushijima et al. showed that *TET* genes and methylation erasers were downregulated in mice with gastric inflammation, causing aberrant methylation [34]. Thus, the mutation spectrum of *TP53* in East Asia, specifically G:C > A:T at CpG sites in GC, reflects the common infectious status of the stomach there. The increased ratio of G:C > T:A in GC in East Asian countries (not statistically significant) may also reflect increased oxyradical DNA damage caused by continued inflammation in the stomach. Non-environmental mechanisms may also play a role in the process. A considerable number of G:C > A:T mutations were also found in Eastern European cases, and the issue of whether G:C > A:T mutations at CpG and non-CpG sites are related to the histological type of GC is still an enigma.

The implication of the difference in *TP53* mutations at CpG and non-CpG sites reminds us of several aspects of environmental gastric carcinogenesis [19]. The epidemiological survey in the cohort by Palli included the diet history of the subjects, which showed that patients with GC with *TP53* mutations at non-CpG sites had a more traditional dietary history, including nitrite, protein, and fat, particularly from animal sources, than in those with mutations at CpG sites. In addition, nitric oxide induced by gastritis may be used to produce *N*-nitroso compounds [35]. *N*-Nitroso compounds can also be taken up by the human body through water, drugs, cosmetics, and tobacco. Since a successful experimental model of GC using *N*-nitroso compounds has been established [36], many investigators have sought evidence of human GC being caused by *N*-nitroso compounds [37]. *N*-Nitroso compounds can generate alkylated guanine adducts, which contribute to G:C > A:T transitions. In contrast, deamination after the nitrosation of guanine and adenine produces xanthine and hypoxanthine, respectively. Hypoxanthine induces A:T > G:C transitional mutations [38]. These adducts may be the first step in human gastric carcinogenesis [39]; however, determining whether the G:C > A:T mutations at non-CpG sites of GC in Eastern Europe involve diet-related *N*-nitroso compounds remains a challenge. The generation of DNA adducts has been attributed to alkylating agents hypothetically [40–42]; however, definitive evidence of the presence of alkyl adducts in the human stomach is still unavailable.

The mutation spectrum of *TP53* in understudied populations may encourage us to pursue the etiological varieties of GC in populations worldwide. We were not able to explain the exact causes of A:T > G:C in GC samples from East Asia. Hongyo et al. have already shown this mutation spectrum in their summary tables and stated that A:T > G:C mutations are prevalent in “Oriental” regions but did not expound much on this finding [20]. Lee DH reported that the metabolites of butadiene, an industrial chemical, including 1,3-butadiene, induced A:T > G:C mutations, exon deletion, and G:C > A:T mutations at the *HPRT* locus in CHO-K1 cells [43]. Some environmental alkyl adducts, which are environmental carcinogens and toxicants [44], may also be involved in A:T > G:C mutations. However, detailed information about the impact of these compounds as possible causes of GC has not yet been provided. Several mutagenic adenine modifications are known; some of them were detected in the gastric mucosa of human GC subjects [40–42]; however, the origins of exposure, the exact chemical process in human tissue, including the production of the intermediates, and the consequences presenting as mutation spectra have not been described yet.

Our study has several obvious limitations. First, we only studied *TP53* mutations, and the sample size was smaller than that generated by the international consortium. As such, hundreds of tumors from Eastern European residents were not compared with those from East Asian residents. It is necessary to confirm our findings by analyzing a large-scale sample set. Second, the designated “Chinese” samples originated from a single institution (Lujiang People Hospital) only; thus, generalizing our findings in this population for Chinese patients with GC would be inappropriate, considering the extensive variations in environmental exposures for these patients. Third, the FFPE quality may not be perfectly controlled. No central pathological diagnosis was made. Primer coverage was not the same; thus, the detectability of splice site mutations may have differed. Currently, data on somatic mutations in human cancers using next-generation sequencing are accumulating, and the implications of our results may need to be re-evaluated. The mutation spectrum of *ARID1A*, which is currently the most prevalent mutated gene in all cancers, is also of interest.

Another problem is the subjective bias of histological typing of GC in these three regions. G:C > A:T mutation prevalence was high (69.6%), especially in the diffuse type of GC in Eastern Europe, in which the ratio of G:C > A:T at non-CpG sites was significantly high (30.3%) (*p* = 0.04 in the two groups, East Europe vs. Asian countries). This apparent difference in the mutation spectrum in different histological subtypes is interesting; however, we must be careful in accepting this finding because we did not use a centralized pathological diagnosis system in this study. In each region, the numbers of blocks that were pathologically investigated were very different, and the method of histological subtyping differed among pathologists from each region. A more comprehensive approach, such as Massive Parallel Sequencing accompanied by centralized pathological assessment, will yield greater information.

We can discuss *TP53* mutations at CpG and non-CpG sites by parsing each mutation signature. Among these mutation signatures, SBS2, presumably associated with activated APOBECs, prefers TpCs. APOBEC functions as an intrinsic off-target deaminase under normal conditions [45]. The consensus target sequences of APOBECs are WpRpC (W = A or T, R = A or G) and TpC, especially TpCpW > TpTpW or TpCpW > TpGpW. The C to T transition at the TpC may explain the G:C > A:T transition in GC in East Asia. Another signature, SBS11, which targets NpCpC/T, is also associated with temozolomide, an anti-cancer alkylating agent. We do not have a demonstrated example of cancers caused by exogenous alkylating agents in natural settings. However, this also rationalizes the pursuit of environmental procarcinogens in the human stomach.

Mutational signature analysis in various populations did not reveal SBSs associated with environmental mutagenic agents. T > C substitutions tended to be higher in China and Japan than in Eastern Europe. Moody et al. reported that the *TP53*-mutation spectrum in alcohol drinkers with esophageal squamous cell carcinoma revealed enrichment of mutations with the characteristic profile of SBS16 compared to the spectrum in non-drinkers [46]. SBS16 had a higher percentage of T > C substitutions than the other substitutions. The difference in the percentages of T > C substitutions between Eastern Europe and East Asia may be due to differences in drinking habits. Genetic variants of ALDH2 and CYP2E1 are involved in alcohol metabolism, and the frequency of these variants differs between Caucasians and Asians [47, 48]. These variants may contribute to the differences in somatic mutation profiles between Eastern Europe and East Asia [47, 49]. Again, the number of substitutions in our study was not sufficient to yield a mutation signature; thus, our interpretation must be handled carefully.

Currently, in many parts of the world or particular accident settings [50, 51], the only available data would be from pathology archives, and a *TP53* mutation spectrum search would still be the most feasible and economical way to speculate the carcinogenesis process. The introduction of cancer gene panels in oncology practice in more areas, including Eastern Europe, may provide more data on these mutation spectra in the future. Expansion of preparations would be necessary to accumulate more extensive data on various geographical characteristics of tumors worldwide. A region-to-region comparison and analyses of different mutation spectra and mutation signatures in populations with different ethnicities, cultures, and habits, as well as of genetic polymorphisms according to ethnicity, may help understand the varied pathways of individual GC carcinogenesis [52, 53].

## Supplementary Information

**Additional file 1: Supplementary Figure S1.** Localization of *TP53* mutations at CpG and non-CpG sites in GC samples from Eastern Europe, China, and Japan (exon 4-8). Mutation-distribution maps were created using the cBioPortal mutation mapper (http://www.cbioportal.org/mutation_mapper). Black dots indicate truncating mutations (nonsense and frameshift mutations). The light purple dot indicates a silent mutation. P53_TAD, TP53 transcriptional activation domain; P53, TP53 DNA-binding domain; P53 tetramer, TP53 tetramer domain.

**Additional file 2: Supplementary Figure S2.***TP53* mutation types in the intestinal and diffuse types of GC samples from Eastern Europe, China, and Japan (exon 4-8). The pie graphs show the percentages of the mutations, including missense (blue), nonsense (orange), and silent mutations (gray), deletions (del) (yellow), deletion-insertion (delins) (light blue), insertions (ins) (light green), and splice site mutations (dark blue) in *TP53* in GC samples from Eastern Europe, China, and Japan. The prevalence of silent mutations (gray) in diffuse-type GCs was significantly different between Europe and Asia (*P* < 0.01). * Statistically significant difference (*p* < 0.05).

**Additional file 3: Supplementary Figure S3.** Mutation spectra in intestinal and diffuse-type GCs in East Europe, China, and Japan. The *TP53* mutations were classified into six types of single nucleotide substitutions, as well as deletions (del), insertions (ins), deletion-insertion (delins), and splice mutations. The G:C > A:T transition was subdivided into G:C > A:T at CpG and non-CpG sites. Each spectrum is shown in the pie graph as follows: G:C > A:T at CpG sites (blue), G:C > A:T at non-CpG sites (orange), A:T > G:C (gray), G:C > C:G (yellow), G:C > T:A (light blue), A:T > C:G (light green), A:T > T:A (dark blue), del (brown), delins (dark gray), ins (light brown), and splice mutations (light navy). The prevalence of G:C > A:T at non-CpG sites (orange) in diffuse-type GCs in Asia was significantly different from that in Eastern Europe (*p* < 0.05). * Statistically significant difference (*p* < 0.05).

**Additional file 4: Supplementary Figure S4.** Designations of populations based on TCGA classification. The majority of the data were from the “White” population. The “Asian” population was not defined, especially whether they were only residing in Asia or not. NA, not available.

**Additional file 5: Supplementary Figure S5.** Mutation spectrum of the *TP53* gene in GC in each population. Six substitution types are depicted in the bar graph as follows: C > A (light blue), C > G (black), C > T (red), T > A (gray), T > C (yellow-green), and T > G (pale orange).

**Additional file 6: Table S1.** Primers for PCR amplification of *TP53* gene. **Table S2.** Histological type and *TP53* mutation status. **Table S3.** Mutation distribution in *TP53* (exon 4–8). **Table S4.** Mutation functions of *TP53* (exon 4-8) in Eastern Europe, China and Japan. **Table S5.** Mutation spectrum of *TP53* (exon 4–8) in Eastern Europe, China and Japan. **Table S6.** Hotspot mutation in Eastern Europe, China and Japan.

## Data Availability

Available on reasonable request.
