# Supplementary figures and images for: Non-CpG sites preference in G:C > A:T transition of TP53 in gastric cancer of Eastern Europe (Poland, Romania and Hungary) compared to East Asian countries (China and Japan)
Source: Genes Environ. 2023 Jan 4;45:1. doi: 10.1186/s41021-022-00257-y (PMC9811704; doi:10.1186/s41021-022-00257-y)

## Slide 1
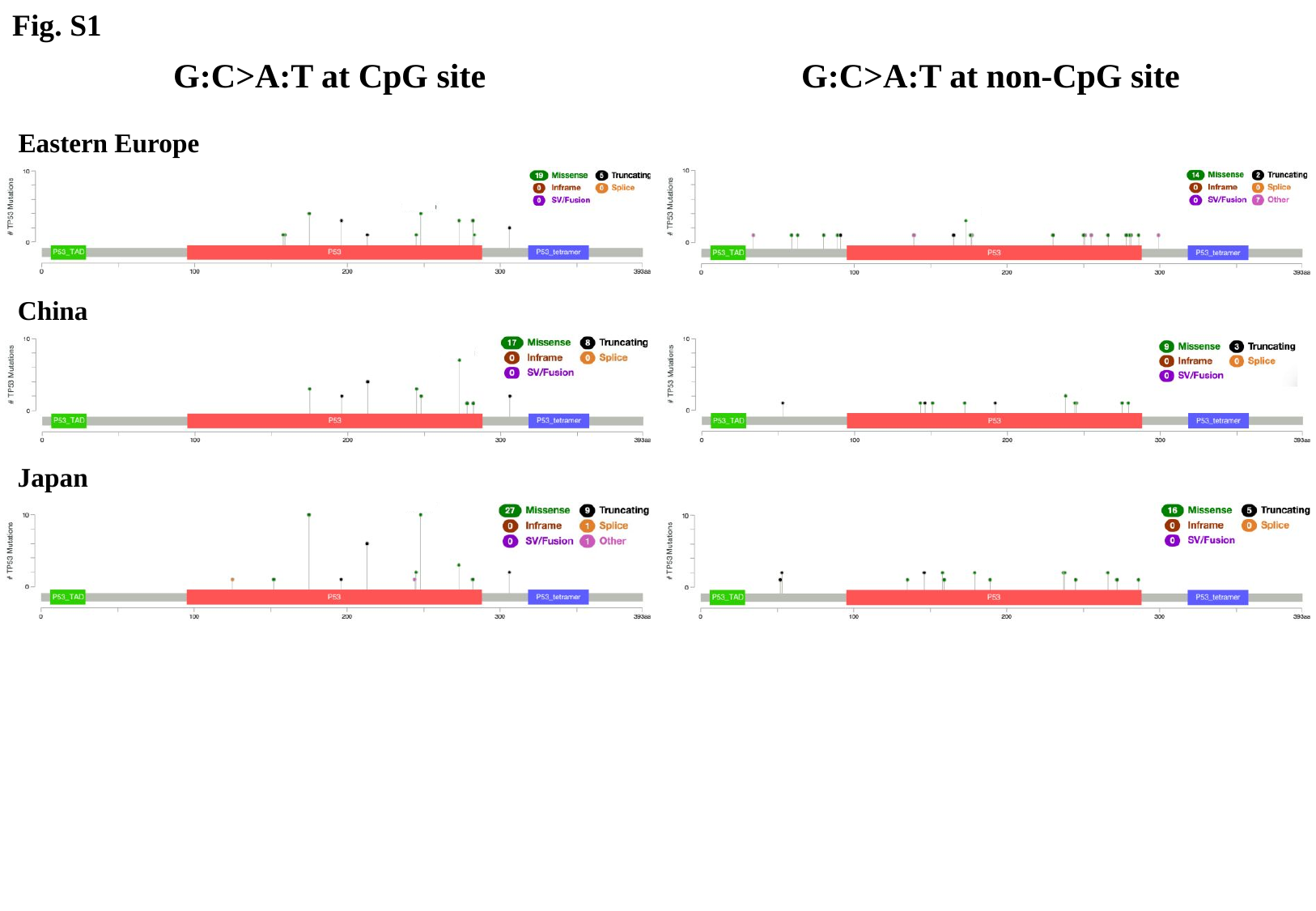

Fig. S1
G:C>A:T at CpG site
G:C>A:T at non-CpG site
Eastern Europe
China
Japan

Supplement: Supplementary file 1 — Additional file 1: Supplementary Figure S1. Localization of TP53 mutations at CpG and non-CpG sites in GC samples from Eastern Europe, China, and Japan (exon 4-8). Mutation-distribution maps were created using the cBioPortal mutation mapper (http://www.cbioportal.org/mutation_mapper). Black dots indicate truncating mutations (nonsense and frameshift mutations). The light purple dot indicates a silent mutation. P53_TAD, TP53 transcriptional activation domain; P53, TP53 DNA-binding domain; P53 tetramer, TP53 tetramer domain. [file 41021_2022_257_MOESM1_ESM.pptx]
