# Supplementary material for: Non-CpG sites preference in G:C > A:T transition of TP53 in gastric cancer of Eastern Europe (Poland, Romania and Hungary) compared to East Asian countries (China and Japan)
Source: Genes Environ. 2023 Jan 4;45:1. doi: 10.1186/s41021-022-00257-y (PMC9811704; doi:10.1186/s41021-022-00257-y)
Supplement: Supplementary file 2 — Additional file 2: Supplementary Figure S2.TP53 mutation types in the intestinal and diffuse types of GC samples from Eastern Europe, China, and Japan (exon 4-8). The pie graphs show the percentages of the mutations, including missense (blue), nonsense (orange), and silent mutations (gray), deletions (del) (yellow), deletion-insertion (delins) (light blue), insertions (ins) (light green), and splice site mutations (dark blue) in TP53 in GC samples from Eastern Europe, China, and Japan. The prevalence of silent mutations (gray) in diffuse-type GCs was significantly different between Europe and Asia (P < 0.01). * Statistically significant difference (p < 0.05). [file 41021_2022_257_MOESM2_ESM.pptx]

## Slide 1
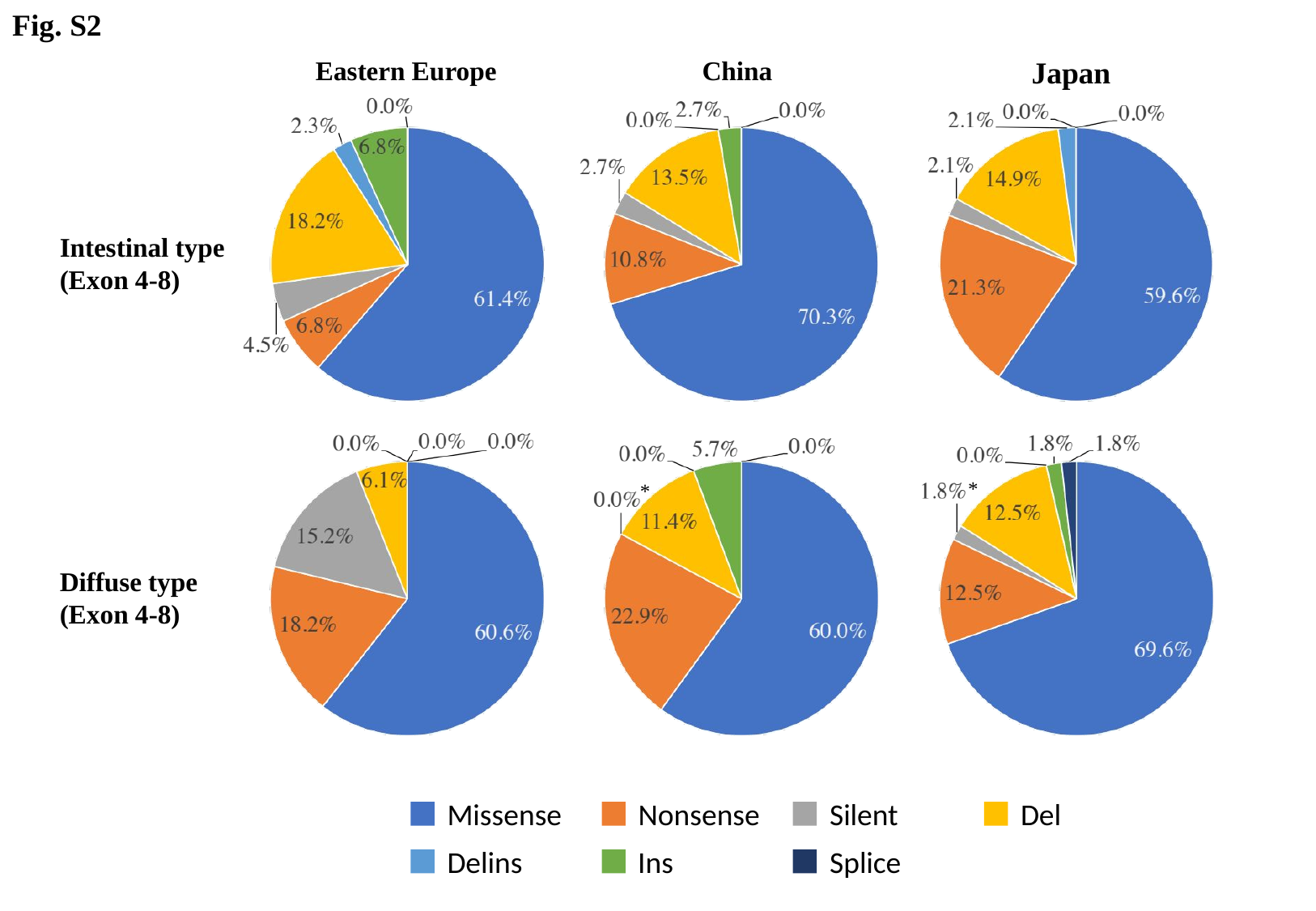

Fig. S2
Eastern Europe
China
Japan
Intestinal type
(Exon 4-8)
*
*
Diffuse type (Exon 4-8)
Missense
Nonsense
Silent
Del
Delins
Ins
Splice
