# Supplementary material for: Non-CpG sites preference in G:C > A:T transition of TP53 in gastric cancer of Eastern Europe (Poland, Romania and Hungary) compared to East Asian countries (China and Japan)
Source: Genes Environ. 2023 Jan 4;45:1. doi: 10.1186/s41021-022-00257-y (PMC9811704; doi:10.1186/s41021-022-00257-y)
Supplement: Supplementary file 3 — Additional file 3: Supplementary Figure S3. Mutation spectra in intestinal and diffuse-type GCs in East Europe, China, and Japan. The TP53 mutations were classified into six types of single nucleotide substitutions, as well as deletions (del), insertions (ins), deletion-insertion (delins), and splice mutations. The G:C > A:T transition was subdivided into G:C > A:T at CpG and non-CpG sites. Each spectrum is shown in the pie graph as follows: G:C > A:T at CpG sites (blue), G:C > A:T at non-CpG sites (orange), A:T > G:C (gray), G:C > C:G (yellow), G:C > T:A (light blue), A:T > C:G (light green), A:T > T:A (dark blue), del (brown), delins (dark gray), ins (light brown), and splice mutations (light navy). The prevalence of G:C > A:T at non-CpG sites (orange) in diffuse-type GCs in Asia was significantly different from that in Eastern Europe (p < 0.05). * Statistically significant difference (p < 0.05). [file 41021_2022_257_MOESM3_ESM.pptx]

## Slide 1
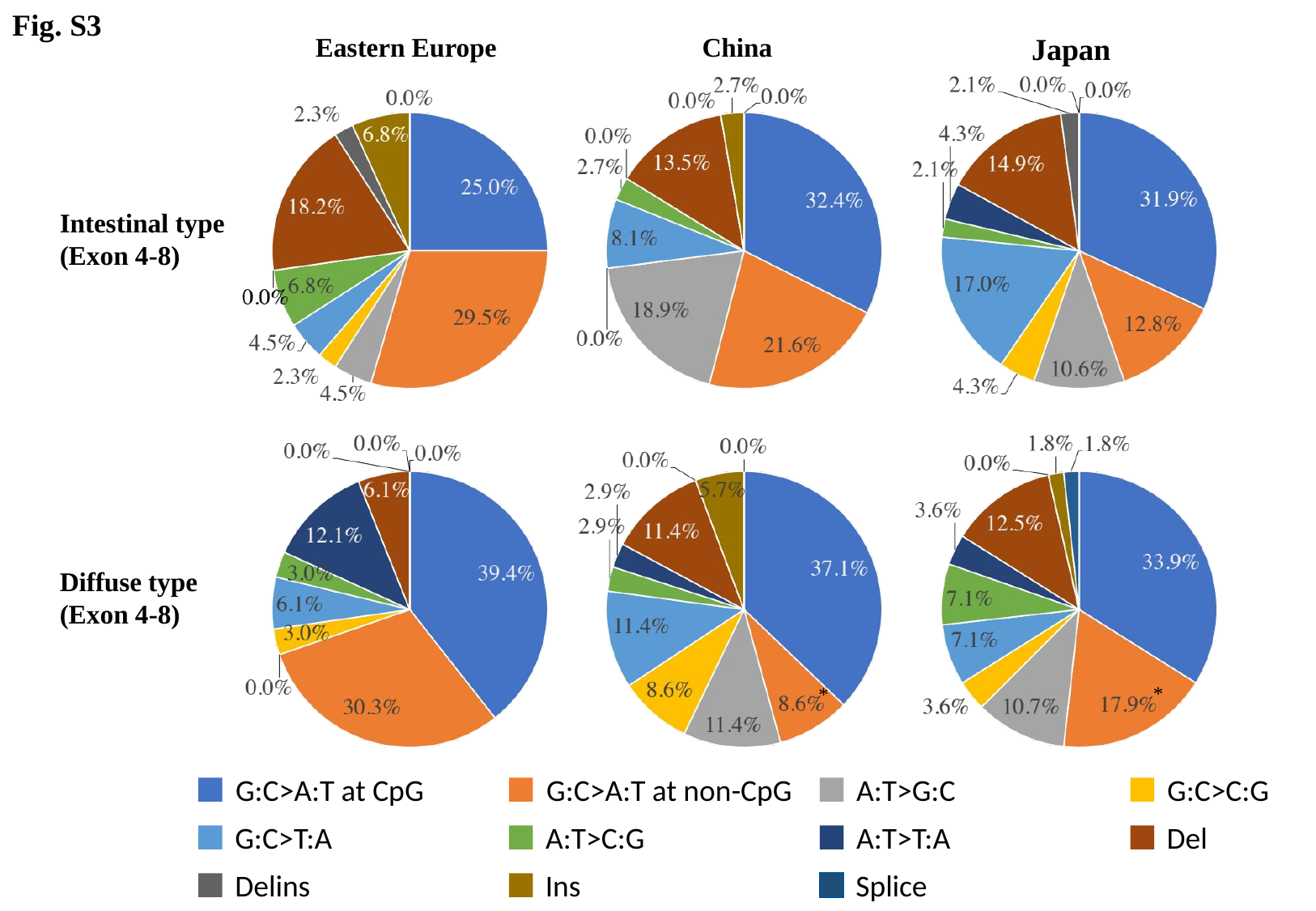

Fig. S3
Eastern Europe
China
Japan
Intestinal type
(Exon 4-8)
Diffuse type (Exon 4-8)
*
*
G:C>A:T at CpG
G:C>A:T at non-CpG
A:T>G:C
G:C>C:G
G:C>T:A
A:T>C:G
A:T>T:A
Del
Delins
Ins
Splice
