# Supplementary material for: Non-CpG sites preference in G:C > A:T transition of TP53 in gastric cancer of Eastern Europe (Poland, Romania and Hungary) compared to East Asian countries (China and Japan)
Source: Genes Environ. 2023 Jan 4;45:1. doi: 10.1186/s41021-022-00257-y (PMC9811704; doi:10.1186/s41021-022-00257-y)
Supplement: Supplementary file 4 — Additional file 4: Supplementary Figure S4. Designations of populations based on TCGA classification. The majority of the data were from the “White” population. The “Asian” population was not defined, especially whether they were only residing in Asia or not. NA, not available. [file 41021_2022_257_MOESM4_ESM.pptx]

## Slide 1
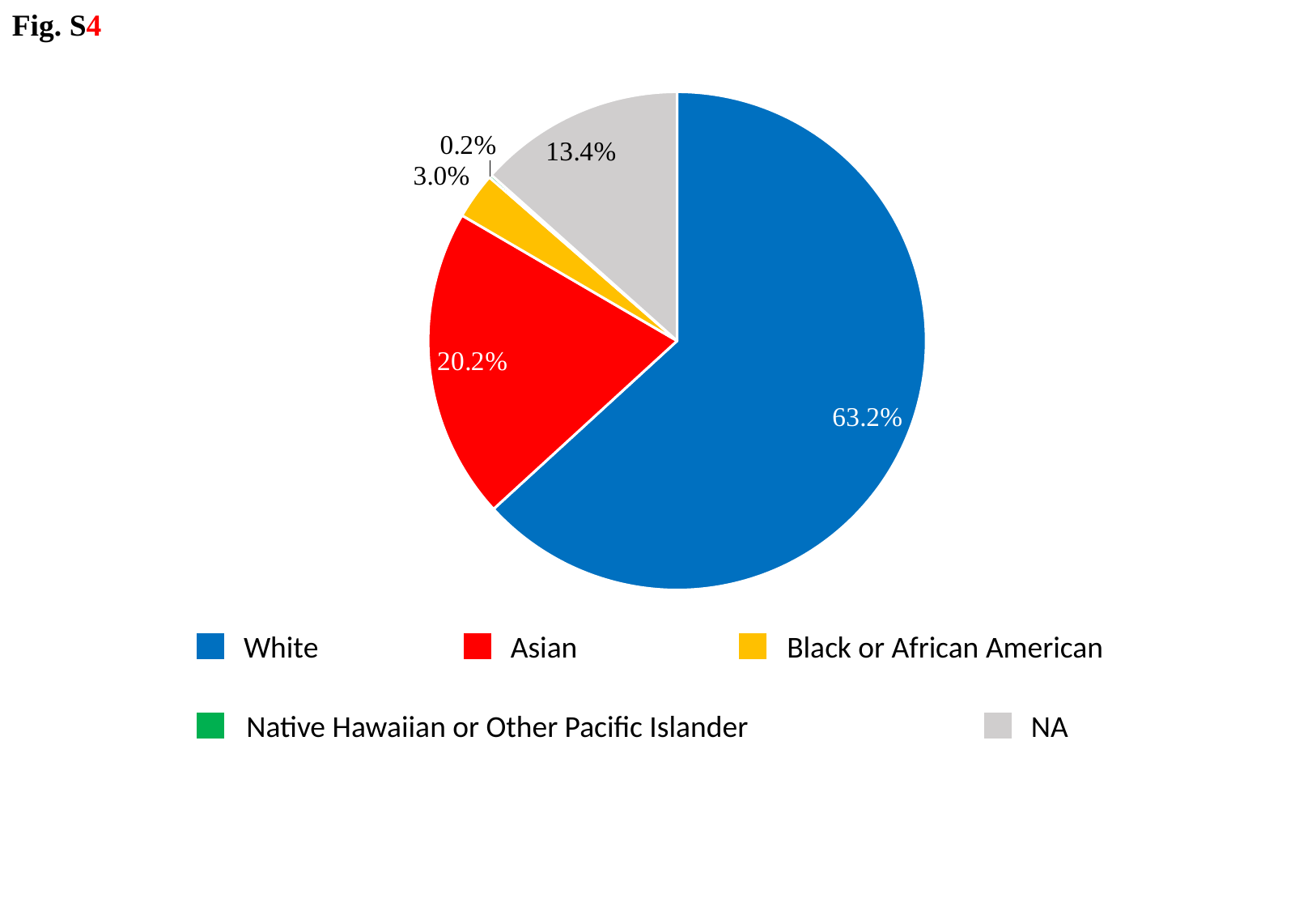

Fig. S4
### Chart
| Category | |
|---|---|
| white | 0.632 |
| Asian | 0.202 |
| Black or African American | 0.03 |
| Native Hawaiian or Other Pacific Islander | 0.002 |
| NA | 0.134 |White
Asian
Black or African American
Native Hawaiian or Other Pacific Islander
NA
