# Supplementary material for: Non-CpG sites preference in G:C > A:T transition of TP53 in gastric cancer of Eastern Europe (Poland, Romania and Hungary) compared to East Asian countries (China and Japan)
Source: Genes Environ. 2023 Jan 4;45:1. doi: 10.1186/s41021-022-00257-y (PMC9811704; doi:10.1186/s41021-022-00257-y)
Supplement: Supplementary file 5 — Additional file 5: Supplementary Figure S5. Mutation spectrum of the TP53 gene in GC in each population. Six substitution types are depicted in the bar graph as follows: C > A (light blue), C > G (black), C > T (red), T > A (gray), T > C (yellow-green), and T > G (pale orange). [file 41021_2022_257_MOESM5_ESM.pptx]

## Slide 1
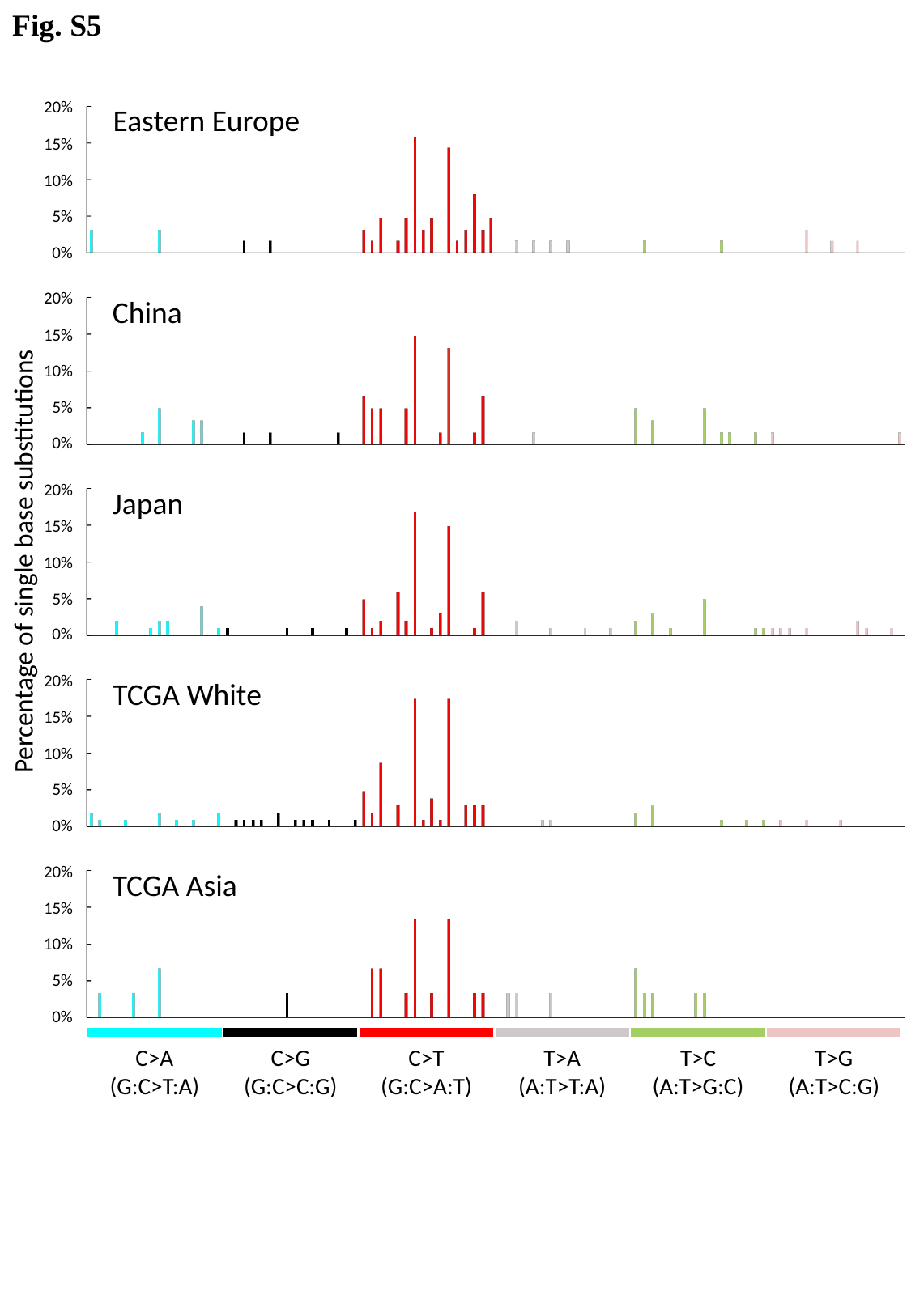

Fig. S5
20%
Eastern Europe
15%
10%
5%
0%
20%
China
15%
10%
5%
0%
20%
Japan
15%
Percentage of single base substitutions
10%
5%
0%
20%
TCGA White
15%
10%
5%
0%
20%
TCGA Asia
15%
10%
5%
0%
C>A
(G:C>T:A)
C>G
(G:C>C:G)
C>T
(G:C>A:T)
T>A
(A:T>T:A)
T>C
(A:T>G:C)
T>G
(A:T>C:G)
